# Supplementary figures and images for: Intercalation of small molecules into DNA in chromatin is primarily controlled by superhelical constraint
Source: PLoS One. 2019 Nov 20;14(11):e0224936. doi: 10.1371/journal.pone.0224936 (PMC6867626; doi:10.1371/journal.pone.0224936)

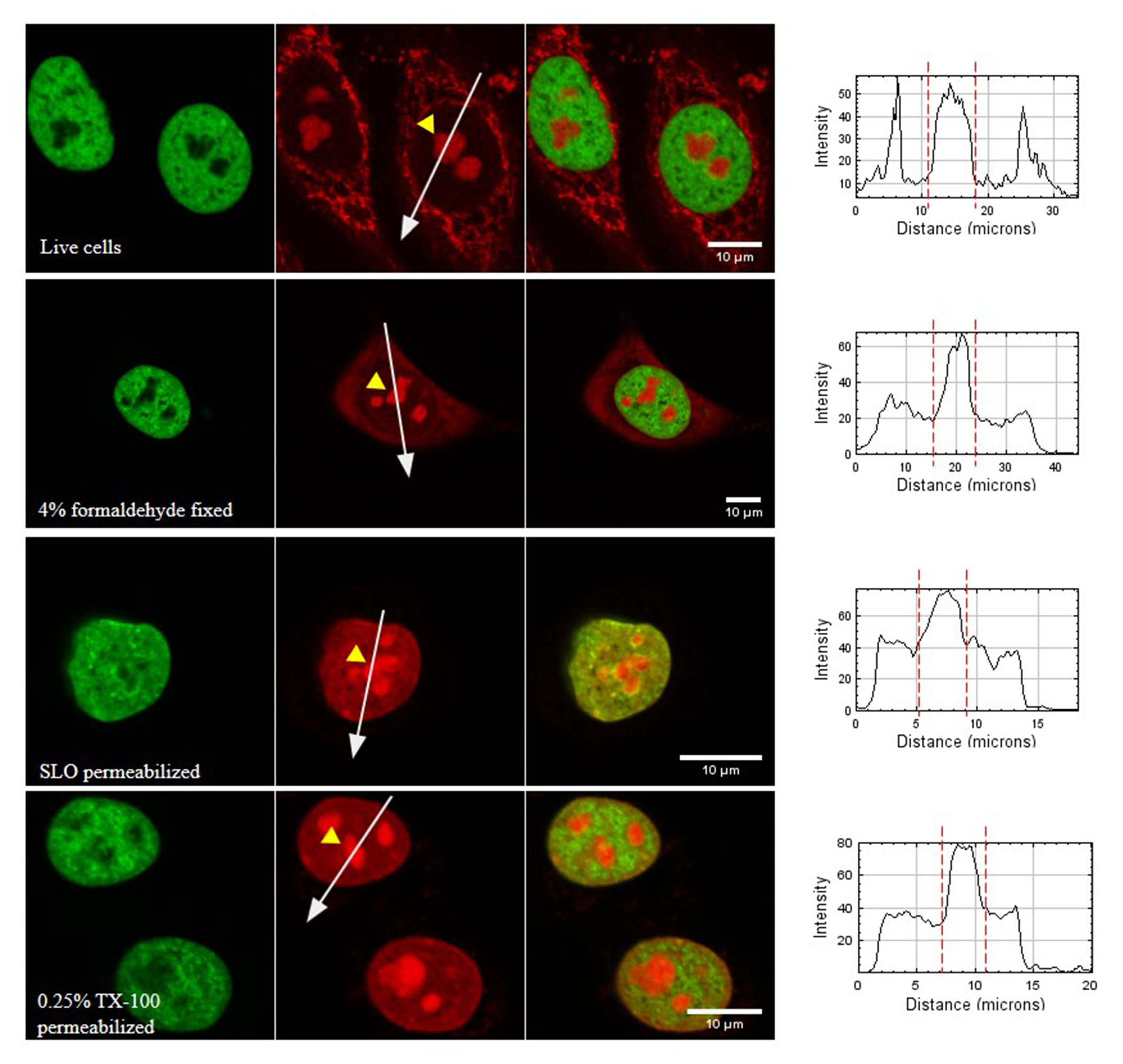

Supplement: S1 Fig — The plots on the right show the EBr fluorescence line scans in the direction of the white arrows in the corresponding image on the left. Yellow arrow heads point at representative nucleoli falling between the red dashed lines on the line scans. Treatments are indicated on the respective panels. EBr fluorescence, red; GFP fluorescence, green. (TIF) [file pone.0224936.s001.tif]

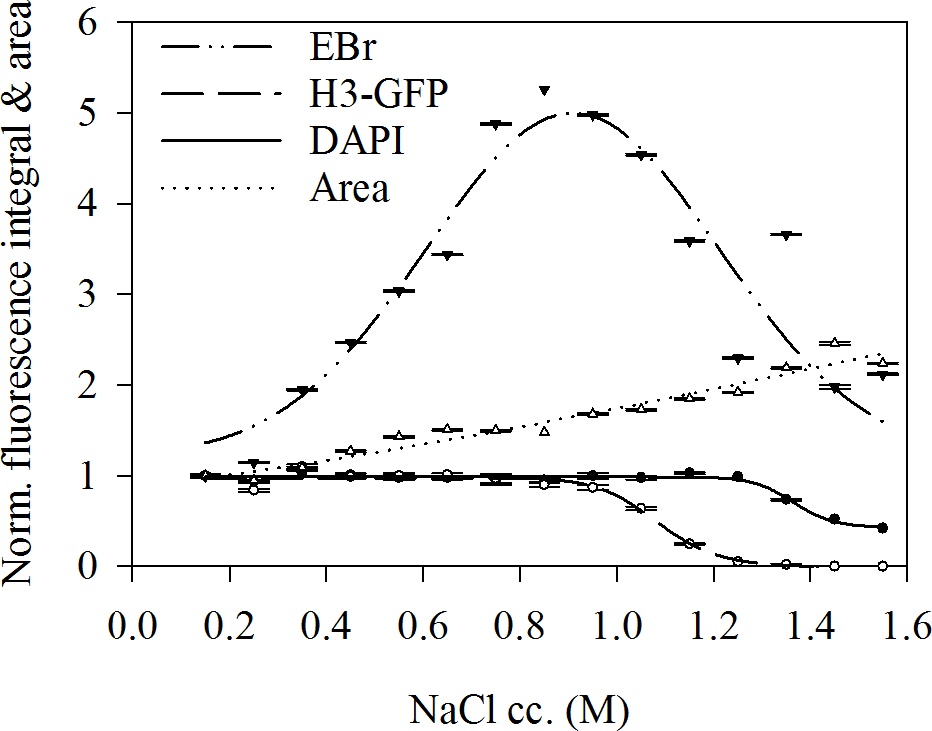

Supplement: S2 Fig — Agarose embedded, salt pretreated HeLa-H3-GFP nuclei. (TIF) [file pone.0224936.s002.tif]

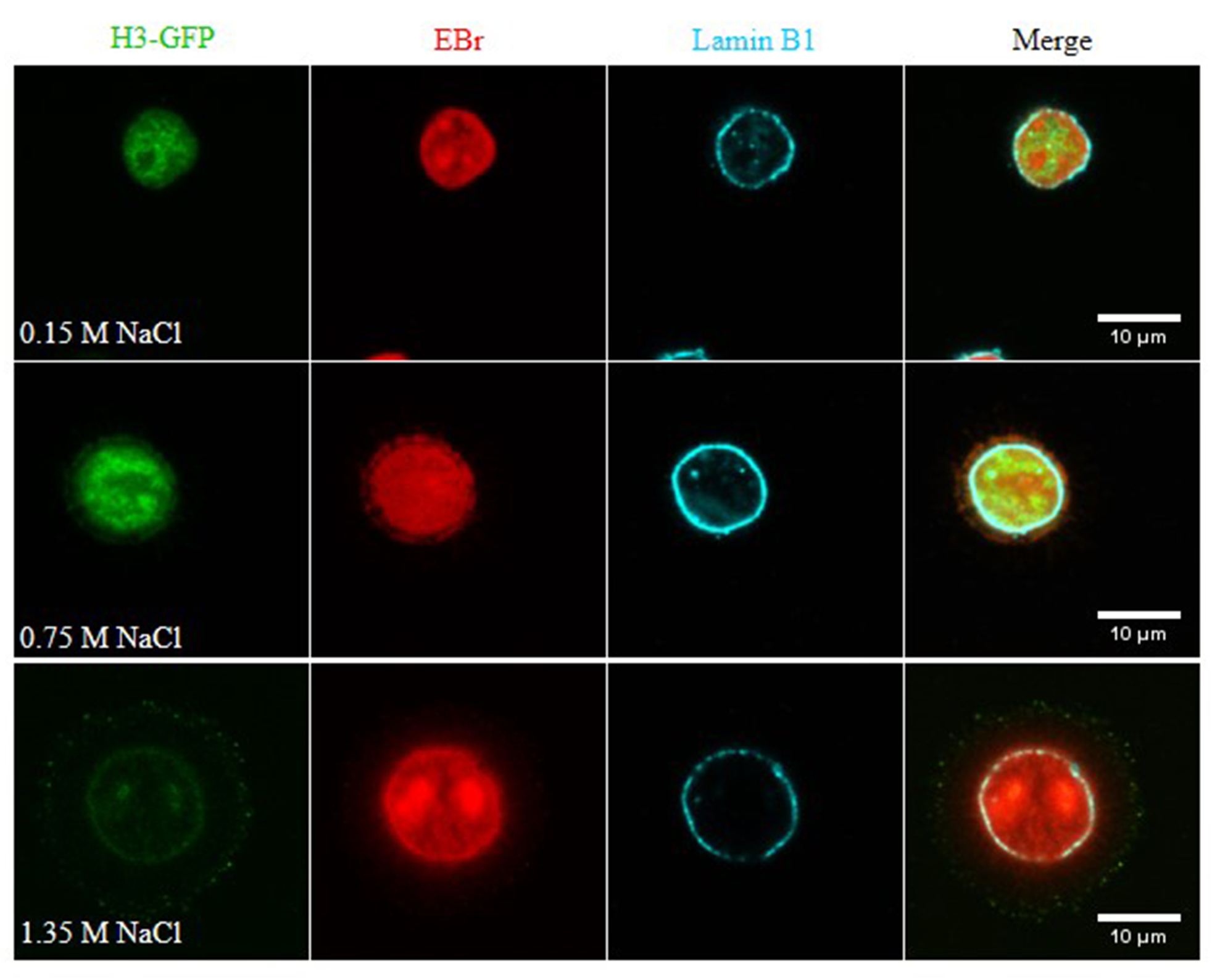

Supplement: S3 Fig — Representative confocal microscopy images of salt treated agarose embedded HeLa- H3-GFP nuclei. Salt concentration is indicated in each panel EBr fluorescence, red; GFP fluorescence, green, Lamin B1 Cyan. (TIF) [file pone.0224936.s003.tif]

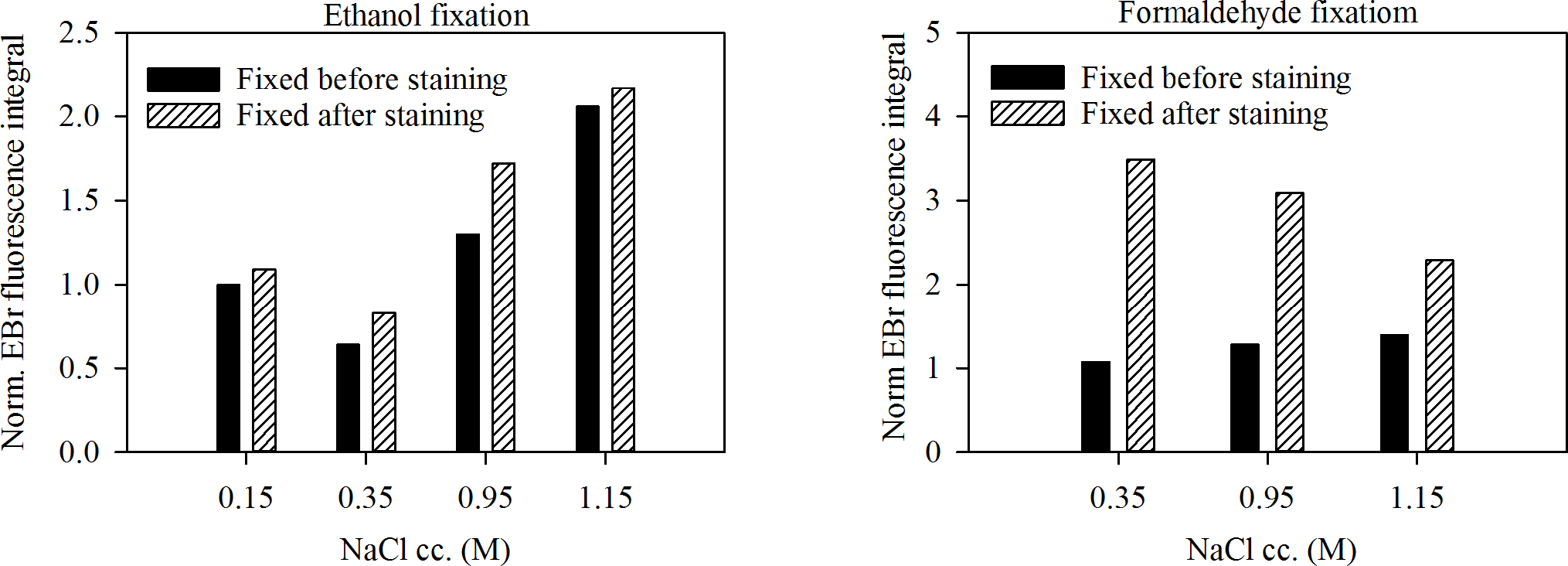

Supplement: S4 Fig — The columns show the normalized mean EBr fluorescence of ~750 nuclei measured by LSC. (TIF) [file pone.0224936.s004.tif]

X- ray dose (Gy)

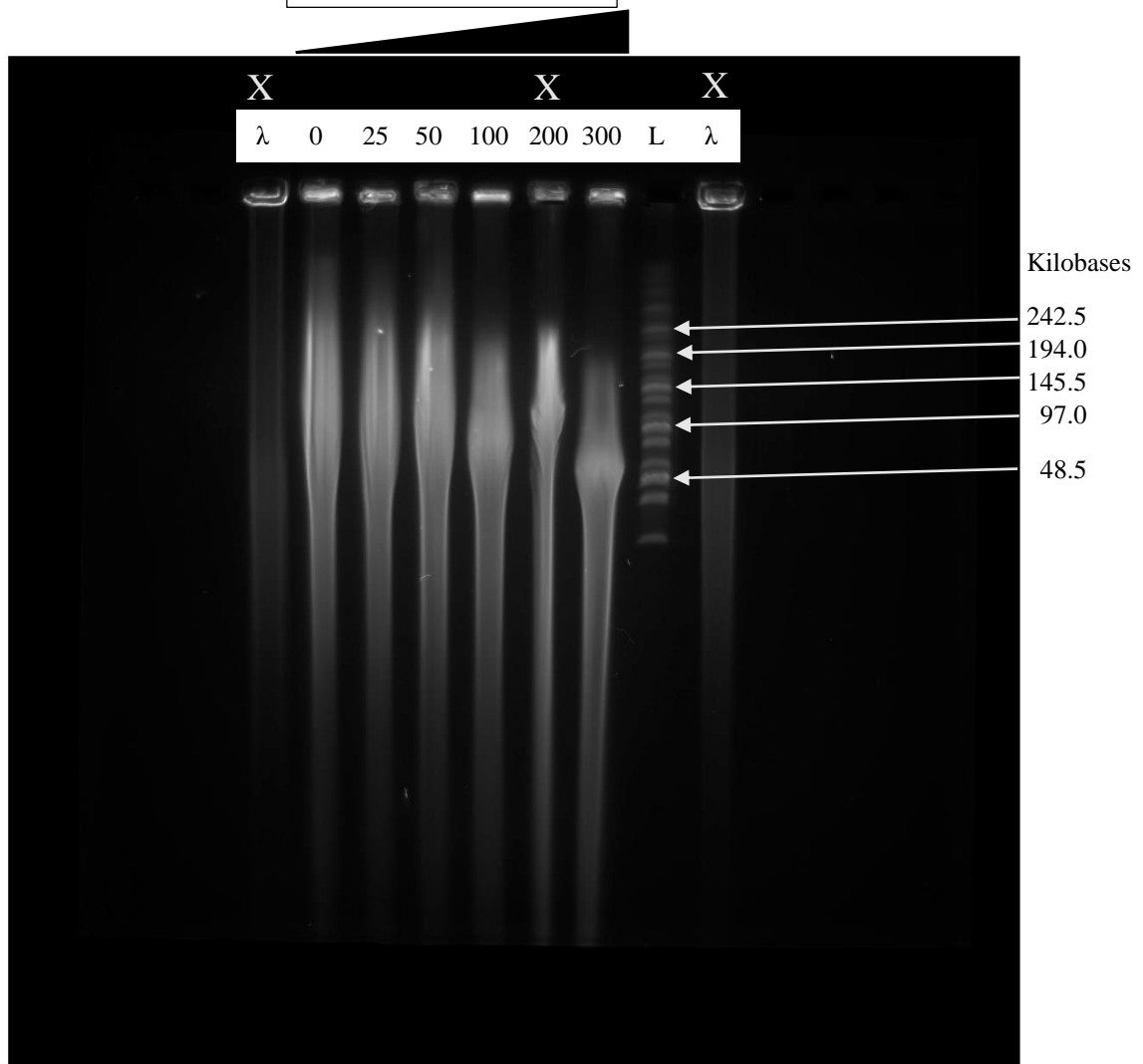

Supplement: S1 Raw Image — The nicks were converted to double strand DNA breaks by S1 nuclease digestion. The DNA samples were analysed on agarose gels by CHEF electrophoresis. Gel was stained with 0.5 μg/ml ethidium bromide and imaged using FluorChem Q (Alpha Innotech, San Leandro, California, USA) gel documentation system; λ-Lambda DNA, L; Molecular Weight Marker (Midrange PFG marker New England Biolabs N0342S). Lanes marked by an X were excluded from the final image in Fig 5B. (PDF) [file pone.0224936.s005.pdf]
