# Supplementary material for: Intercalation of small molecules into DNA in chromatin is primarily controlled by superhelical constraint
Source: PLoS One. 2019 Nov 20;14(11):e0224936. doi: 10.1371/journal.pone.0224936 (PMC6867626; doi:10.1371/journal.pone.0224936)
Supplement: Attachment — Submitted filename: Response to Reviewers.docx [file pone.0224936.s006.docx]

**Answers to Reviewers’ comments**

**Reviewer 1**

We thank Reviewer for the overall positive evaluation of our work. We also greatly appreciate Reviewer’s comments and suggestions which helped us improve the quality and depth of the manuscript, especially with regard to nucleosome dynamics, an aspect we had not elaborated on in our first version of the manuscript. Below we address the specific remarks and have given the relevant line numbers in the “Revised Manuscript with track changes”.

**1. The title gives somewhat the wrong impression what this paper is about as “nucleosomal and internucleosomal impediments” sound much more microscopic than what is actually done in this study. A somewhat less specific title (e.g. chromosomal impediments) would reflect the content of the study better.**

We have changed the title to its present form so as to avoid the impression that this is a morphological study. Page 1 line 1-2

**2. In addition, some references to more microscopic views of how DNA binding proteins can find access to their target sites would be useful to provide. I am thinking here mainly of Jonathan Widom’s site exposure mechanism. Nucleosomal DNA is in principle accessible through the temporary unspooling of wrapped DNA.**

The Widom paper “Li G, Levitus M, Bustamante C, Widom J. Rapid spontaneous accessibility of nucleosomal DNA. Nat Struct Mol Biol. 2005 Jan 5;12(1):46–53” has been cited in the revised Introduction where we elaborated more clearly what the term accessibility might actually cover at the molecular level. Page 3 line 48-50.

**Do the experiments give any hints that intercalators can also bind to nucleosomal DNA or can they not compete with the histones?**

The nucleosomes pose a very stringent barrier to intercalation according to our observations. That is indeed surprising in view of the dynamics alluded to by the Reviewer. However, at equilibrium the set of histone-DNA bonds sampled by the nucleosome at any particular moment include several contacts which together collectively maintain the negatively superhelical toroid state of the DNA. In other words, from the point-of-view of DNA-topology it doesn’t matter through which specific bonds out of the several possible ones the DNA and the histone octamer are engaged. Here we make reference to “Luger K, Richmond TJ. DNA binding within the nucleosome core. Curr Opin Struct Biol. 1998 Feb; 8(1):33–40”: The DNA is bound to the histone core at 14 points where the minor groove faces the octamer involving a total of 142 hydrogen and several electrostatic bonds. This has been pointed out, with the paper being cited, in Discussion. Page 10 line 231-234; already in the first version.

The fact that EBr binding starts to increase at salt concentrations where the histone dimers are evicted is in line with the general perception that nucleosomes start to become destabilized from the edges where the dimers reside and only then does EBr intercalation into nucleosome-bound DNA become possible.

Early observations determined that about 14 EBr molecules can be accommodated in a nucleosomal particle before the histones are released “McMurray CT, Small EW, van Holde KE. Binding of Ethidium to the Nucleosome Core Particle. 2. Internal and External Binding Modes. Biochemistry. 1991; 30(23):5644–52.” Binding of these dye molecules may be allowed by the dynamic structure of the nucleosome pointed out by Reviewer. On the other hand, the superhelical constraints in the reconstituted/isolated nucleosomes used in the early studies may not be as stringent as they are in their native environment approximated better in our assays. Indeed, our MNase data suggest that the levels of EBr staining in low salt conditions, i.e. when all the nucleosomes are in place, is likely due to the binding of the dye to internucleosomal linker DNA (and NFRs) rather than to nucleosome-bound DNA.

**3. Page 3, line 45: The authors write: “Interpretation of accessibility is not obvious, as the DNA is wound around the nucleosome, readily exposed to the environment.” Do the authors refer here to the site exposure? Maybe here they could cite corresponding papers.**

We agree with the Reviewer that access of regulatory molecules to the DNA can be controlled by the time-window when a particular segment of the DNA involved in binding becomes available to engage with the regulator in view of the dynamics of the structure. Indeed, this aspect has not been properly addressed in the first version. Now we have expanded on this aspect in Introduction, as mentioned also above. Also we have included a reference for the ready access to the DNA grooves for DNA wrapped around the nucleosome. Page 3 line 48-50.

**4. Page 4, line 73: The authors mention psoralen here for the first time, without explaining what it is. Only on page 6 it is mentioned that it is an intercalating drug.**

The citation of a reference and an explanatory phrase is included now where the compound is first mentioned. Page 4 line 77-78.

**5. The following lines are hard to understand when reading the manuscript for the first time (but perfectly clear when reading it the second time). Maybe passages like “harbors a level of nucleosome-unconstrained torsion” etc. could be written more clearly.**

The sentence has been reworded so as to improve its clarity. Page 4 line 83.

**6. The Results section starts with “We observed that the chromatin of live cells does not readily stain with the intercalating dye EBr despite its presence in the nucleus shown by the fluorescense of the nucleoli.” When reading this sentence I found completely lost. What experiment is described here? What are we looking at and why? Again, after having read the whole paper, this sentence makes sense (especially when reading it together with the last sentence of the introduction). But for first-time readers I recommend to cut the sentence into several. First explain the experiment. Then, describe what you observe. You might also remove the last sentence of the introduction and incorporate this information into the beginning of the result section.**

We have removed the last sentence regarding “our motivation”. This becomes clear anyway when the first, somewhat reworded, paragraph of Results is read. Page 5 line 103-106

**Reviewer 2**

We thank Reviewer for the overall positive evaluation of our work and for the insightful comments and suggestions which have helped us to improve the quality and depth of the manuscript. We have now discussed the effect of nicking also in light of its possible effects on transcription. Below we address the specific remarks and have given the relevant line numbers in the “Revised Manuscript with track changes”.

**I have only the comment that the intercalation was done in growth medium but the imaging in PBS - can it be excluded that this change of medium affects the binding of ethidium?**

In our initial experiments we stained the cells with EBr in complete medium and observed the dye exclusively in the cytoplasm (due to the DNA of mitochondria) and nucleoli. In the experiment demonstrated in Fig. 1 we actually followed the protocol detailed in the revised Methods, using PBS (i.e. not in complete medium as erroneously described previously). The distribution of the dye in the cells was very similar in both cases, so we do not suspect any major effect of the medium on dye uptake and binding. Page 14 line 323-329.

**Some parts of the presentation could be improved. The title does not present well what appears to be the most interesting finding, that "relaxation by inducing as few as a single nick per ~50 kb enables intercalation in the entire chromatin loop, demonstrating the possibility for long-distance effects of regulatory potential".  In fact, the short title may be more informative**.

In view of the fact that many proteins bind to the DNA involving intercalating moieties, the importance of the observation emphasized by the current title (modified in the wake of Reviewer 1’s suggestion) appears to be quite convincing, while the biological significance of the long-range effects we have observed may not be immediately obvious. Furthermore, including the long-range effects in the title would give the impression that we claim to be the first workers to demonstrate such phenomena. This is not the case in view of the following references: Villeponteau B, Martinson HG. Gamma rays and bleomycin nick DNA and reverse the DNase I sensitivity of beta-globin gene chromatin in vivo. Mol Cell Biol. 1987 May; 7 (5):1917–24. and Kumala S, Hadj-Sahraoui Y, Rzeszowska-Wolny J, Hancock R. DNA of a circular minichromosome linearized by restriction enzymes or other reagents is resistant to further cleavage: an influence of chromatin topology on the accessibility of DNA. Nucleic Acids Res. 2012 Oct 1; 40 (19):9417–28. These references have been cited in the revised version. Page 13 line 292

**In the Discussion, it would be interesting to consider the publication:  Luchnik AN, Hisamutdinov TA, Georgiev GP. 1988. Inhibition of transcription in eukaryotic cells by X-irradiation: relation to the loss of topological constraint in closed DNA loops. Nucleic Acids Res 16:5175–5190. There, "X irradiation was found to inhibit in vivo transcription. About one DNA single-strand break per estimated DNA loop (domain) length is sufficient to explain the effect".  That work suggests that after relaxation, DNA becomes less accessible to the transcriptional machinery, whereas the present study shows that it becomes more accessible to an intercalator.**

We have expanded Discussion so as to point out that topological relaxation leads to an altered chromatin structure (based on the references Villeponteau *et. al* and Kumala *et. al*, stated above) which could account for the decrease of transcription described by **Luchnik** et al. Page 13 line 290-294.

We have also included a deeper explanation of why constrained superhelical twist would hamper intercalation, not specifically asked by Reviewer but felt missing in the wake of this comment. Page 11 line 252-261
